# Characteristics of landing impact in athletes who have not returned to sports at the pre-injury competition level after anterior cruciate ligament reconstruction

**DOI:** 10.1016/j.asmart.2021.05.001

**Published:** 2021-06-04

**Authors:** Shunsuke Ohji, Junya Aizawa, Kenji Hirohata, Takehiro Ohmi, Sho Mitomo, Tetsuya Jinno, Hideyuki Koga, Kazuyoshi Yagishita

**Affiliations:** a Clinical Center for Sports Medicine and Sports Dentistry, Tokyo Medical and Dental University, 1-5-45 Yushima, Bunkyo-ku, Tokyo, 113-8519, Japan; b Department of Physical Therapy, Juntendo University, 3-2-12 Hongo, Bunkyo-ku, Tokyo, 113-0033, Japan; c Department of Orthopaedic Surgery, Dokkyo Medical University Saitama Medical Center, 2-1-50 Minami-Koshigaya, Koshigaya, Saitama, 343-8555, Japan; d Department of Joint Surgery and Sports Medicine, Tokyo Medical and Dental University, 1-5-45 Yushima, Bunkyo-ku, Tokyo, 113-8519, Japan

**Keywords:** Anterior cruciate ligament reconstruction, Return to sports, Athletic performance, Injury

## Abstract

**Background:**

Most patients with anterior cruciate ligament (ACL) injury undergo ACL reconstruction (ACLR) with the expectation of being able to return to sport (RTS) at the same level of the competition as before the injury. The magnitude and asymmetry of landing impact are important post-ACLR functional variables related to increased ACL strain and poor athletic performance. However, the association between the RTS status and landing impact in post-ACLR patients is unknown.

**Objective:**

To investigate the association between RTS status and landing impact during single-leg landing in post-ACLR patients.

**Methods:**

Forty-four patients after primary, unilateral ACLR participated in this study. They had already participated in sports post-ACLR. Questionnaires were used to assess whether the participants achieved the same competitive level of RTS as before the injury. The magnitude and symmetry of the peak vertical ground reaction force (pVGRF) were collected and analysed during single-leg jump landings. Additionally, knee functions (range of motion, laxity, effusion, strength, and single-leg hop distance) were measured.

**Results:**

A total of 28 (64%) patients reported RTS at their pre-injury competition levels. The no-RTS group had a lower pVGRF magnitude on the operated side than the yes-RTS group (*P* = .019). The no-RTS group had a higher rate of pVGRF asymmetry (50%) than the yes-RTS group (18%) (*P* = .040). Logistic regression analysis revealed that pVGRF magnitude and asymmetry were significantly associated with the RTS status. Logistic regression analysis adjusted for knee function revealed that the pVGRF magnitude was significantly associated with the RTS status.

**Conclusion:**

In patients who are unable to RTS at their pre-injury competition level after ACLR, the pVGRF is lower and more likely to be asymmetrical than in those able to RTS at their pre-injury competition level.

## Introduction

Most patients with anterior cruciate ligament (ACL) injury undergo ACL reconstruction (ACLR) with the expectation of being able to return to sport (RTS) at the same level of the competition as before the injury.^1^ However, in a meta-analysis of post-ACLR patients, only approximately 63% of patients were able to RTS at their pre-injury competition level.^2^

Factors related to RTS status after ACLR can be categorized into demographic information such as age, gender and competition level, knee joint function, and psychological factors.^3^ Post-ACLR knee function characteristics, such as greater laxity,^4^ swelling,^5^ weak knee extensor strength,^6^ and greater asymmetry in single-leg hop distance,^7^ have been reported in patients who have not been able to RTS at their pre-injury competition level.

In patients after ACLR, the magnitude and asymmetry of landing impact are important functional variables related to increased ACL strain and poor athletic performance such as jump and change of direction.^8^^,^^9^ In a previous study comparing the landing impact of patients after ACLR and healthy athletes, the peak vertical ground reaction force (pVGRF) on the operated side in patients after ACLR was less than that in healthy athletes.^10^ In previous studies comparing pVGRF during landing in patients after ACLR on the operated and nonoperated sides, pVGRF on the operated side was lower and more likely to be asymmetrical.^11^, ^12^, ^13^

The pVGRF during single-leg landings is larger than that in other jump landing tasks.^14^ Since most ACL injuries occur in single-leg landings rather than double-leg landings,^15^ it is important to analyse the biomechanics during single-leg landings.^16^ Therefore, this study aimed to investigate the association between the RTS status and landing impact during landings in post-ACLR patients. Additionally, the strength of the association between RTS and landing impact was evaluated while accounting for knee function variables. We hypothesized that patients who are unable to RTS at their pre-injury competition level would have a lower and more asymmetrical pVGRF on the operated side than those able to RTS at their pre-injury competition level, even after adjusting for knee function variables.

## Materials and methods

### Participants

Participants who had undergone primary ACLR between April 2012 and May 2018 and already participated in sports post-ACLR were included if they met the following inclusion criteria: 16–45 years old at the time of questionnaire administration; participation in sports with a modified Tegner activity scale^17^ score ≥5 before injury; and 8 months or more after surgery. Participants were excluded if: they had not participated in sports for social reasons such as pregnancy and employment; they had ACL injury to the contralateral knee or ACL re-injury to the reconstructed knee; they had a history of meniscal injury and surgery of the ipsilateral knee and the contralateral knee before ACL injury; they had a cartilage injury requiring surgery; they had difficulty in follow-up until RTS; and they had a complication that would interfere with their RTS after surgery. These were the same participants and selection procedures as previously reported.^18^

### Surgical technique and post-operative rehabilitation

All surgeries were performed by orthopaedic surgeons specialized in knee joint. The autograft sources were hamstrings or bone-patellar tendon-bone. Postoperative rehabilitation protocol was based on the previous study.^18^ Range of motion (ROM) and quadriceps isometric contraction exercises were started three days after surgery. A straight-position knee-joint immobilizer (Knee brace, ALCARE Co., Ltd., Tokyo, Japan) and crutches were used and then gradually phased out from four weeks after ACLR. Jogging started three months after ACLR and the running speed was gradually increased. Sports participation was allowed by a doctor when the following were achieved: it was at least six months after surgery; limb symmetry index (LSI) of isokinetic knee extension and flexion torque was >85%, as measured with a BIODEX System 4 Isokinetic Dynamometer (BIODEX Medical Inc., Shirley, NY) at 60°/s and 180°/s); and LSI on the single-leg hop distance was >90%. The postoperative rehabilitation protocol was the same for all patients. However, patients who underwent repair of the middle-posterior segment of the meniscus were prohibited from deep squatting or bending the knee more than 90° until three months after surgery.^18^

### Procedures

This was a cross-sectional study completed in a single centre. Demographic, injury, and operated information were collected from medical records. Participants' height, weight, knee functions, landing impact and questionnaire were measured on the same day. Ethical approval was obtained from the Ethics Committee (approval number: M2016-197). All participants provided written informed consent prior to participation.

### Measurement of knee functions

Knee functions, such as ROM, effusion, anterior knee laxity, muscle strength, and single-leg hop distance, were measured. These evaluations have moderate-to-high intra-rater and inter-rater reliabilities.^19^, ^20^, ^21^, ^22^ Knee flexion and extension ROM were measured in increments of 1° using a goniometer. The angle obtained by subtracting the operated side's value from the value on the non-operated side was defined as the ROM deficit.

Effusion was assessed using the stroke test,^20^ which evaluates the degree of effusion on a 5-point scale (zero, trace, 1+, 2+, 3+). Higher scores indicate a greater degree of effusion.

Anterior knee laxity was measured using KT-1000 (MEDmetric Corp., San Diego, CA) at the maximum manual pull and expressed as the difference between the non-operated and operated sides.^23^

Isokinetic knee strength was assessed using Biodex System 4. Extensor and flexor peak torques were assessed at angular velocities of 60°/s and 180°/s, respectively. The peak torque was expressed in Nm divided by the body weight (Nm/kg). Additionally, the limb symmetry index (LSI) was calculated as the ratio of the operated and non-operated sides’ peak torques.^24^

Single-leg hop distance was measured as a functional test. It is a functional test to evaluate lower extremity power during hopping and dynamic stability during landing by measuring the hop distance.^25^ Participants stood on one leg behind the starting line, hopped forward as far as possible, and landed on the same leg. A stable landing meant a successful test. The definition of an unsuccessful hop was an additional hop upon landing, landing with premature touchdown of the contralateral limb, and/or loss of balance.^26^ The participant's arm movements during jump landing were not restricted. Each participant performed three practice jumps, followed by two successful trials. The longest distance (cm) of the trials was recorded, and the LSI was also calculated.

### Landing impact during single-leg jump landing on a force plate

All participants performed a single-leg jump landing task.^27^ A 20-cm-high box was placed 60 cm from the centre of the force plate. The participants stood on the step on their measurement leg with the opposite hip and knee bent at 45° and 90°, respectively, and neutral hip rotation. The arms were crossed to eliminate the effect of their movement. Participants jumped forward without intentional upward action and landed naturally on the force plate's centre and maintained balance for 5 s. No specific instructions about landing were provided, and the participants performed the task according to their preference. A trial was deemed unacceptable if the arms left the chest, the foot fell outside the force plate at landing, the foot moved or slid after landing, or the sole of the opposite foot touched the force plate or the floor. Each participant performed least three practice jumps, followed by three successful trials as a formal test.

VGRF was recorded during landing using a force plate (260AA6, Kistler Instrument AG, Winterthur, Switzerland). The sampling rate was 1000 Hz, and the VGRF data were filtered through a fourth-order Butterworth low-pass digital filter at a cutoff frequency of 50 Hz and normalized by body weight using a software (IFS-4J/3J, DKH). The pVGRF data were calculated as pVGRF magnitudes. The average of the three trials was calculated. Measurement of the VGRF during single-leg jump landing has demonstrated acceptable test-retest reliability.^28^

Exceeding 100% is not always a good result for the pVGRF's LSI during single-leg jump landing.^24^ Thus, the absolute symmetry index (ASI), which represents the absolute value of the asymmetry between the operated (S) and non-operated sides (N), was calculated as follows:

$$ASI\;\left(\%\right)=\left|\frac{S-N}{0.5\times \left(S+N\right)}\right|\times 100$$

An ASI > 10% was defined as asymmetry.^29^

### RTS status

All participants answered two questions. The dichotomous (yes/no) question was, “Have you returned to the same level of competition as before your ACL injury?“^6^ The continuous-response (0%–100%) question was, “What is the subjective performance intensity of the sport you are currently participating in?” The latter question is an index of postoperative subjective athletic performance (PoSAP), and 100% means that the current performance intensity is 100% of the subjective athletic performance before the ACL injury. Ohji et al.^18^ reported that most post-ACLR patients with a PoSAP of 80% responded “Yes” to the dichotomous question and that the dichotomous question alone tended to over evaluate their RTS status. Therefore, in this study, the participants who answered “Yes” to the dichotomous question and >80% for the PoSAP were included in the yes-RTS (YRTS) group. The no-RTS group included those who met none or only one of the criteria.

### Statistical analysis

The normality of each variable's distribution was determined using the histogram and the Shapiro–Wilk normality test. Means and standard deviations were calculated for normally distributed variables, whereas medians (quartile range) were calculated for variables that are not normally distributed.

The differences in demographic data, knee functions, and landing impact between the NRTS and YRTS groups were analysed using the chi-square test, Fisher's exact test, unpaired *t*-test, or Mann–Whitney *U* test. Additionally, effect sizes (chi-square test = ϕ coefficient, Fisher's exact test = Cramer's V, *t-*test = Cohen's d, Mann–Whitney *U* test = r) were calculated for each variable.

The associations between landing impact (pVGRF magnitude and asymmetry) and the RTS status were examined using logistic regression analysis (forced entry method). First, the analysis was performed with pVGRF magnitude and asymmetry as independent variables and then adjusted for variables with *P* < .1 in the between-group comparisons. The *a* priori α level was set at 0.05. Data were analysed using SPSS version 21.0 (IBM Corp, Armonk, NY, USA).

## Results

Forty-four patients after ACLR were included in this study. Sixteen (36%) and twenty-eight (64%) patients were assigned to the NRTS and YRTS groups, respectively. One patient responded “Yes” to the dichotomous question but had a PoSAP ≤80%, and one responded “No” to the dichotomous question but had a PoSAP >80%. The lowest PoSAP for the YRTS group was 85%.

The demographic characteristics are summarized in Table 1. Patients in the NRTS group had higher body mass index (BMI) values than those in the YRTS group (*P* = .032, *d* = −0.32). The results of Fisher's exact test showed that the NRTS group (44%) had a higher proportion of overweight (BMI ≥ 25 kg/m^2^) than the YRTS group (11%) (*P* = .017, V = .38). There were no significant differences in other demographic characteristics, operated information, or activity level between the two groups.

**Table 1:** Demographic characteristics of the study participants.

Variables	NRTS (n = 16)	YRTS (n = 28)	*P* value	Effect size
PoSAP^a^	72.5 (17.5)	100.0 (8.7)		
Age, y^a^	23.0 (13.0)	21.0 (5)	.185	-.20
Sex (female/male), n	4/12	15/13	.113	.23
BMI^a^	23.9 (4.9)	22.1 (2.9)	.032∗	-.32
BMI ≥ 25, n (%)	7 (44%)	3 (11%)	.017∗	.38
Injury type (contact/non-contact), n	3/13	5/23	.620	-.01
Surgery to measurement, m^a^	15.5 (10.8)	23.5 (21.0)	.187	.14
Graft type (ST/BTB), n	14/2	26/2	.614	.09
Meniscus repair (yes/no), n	10/6	22/6	.303	.17
Pre-injury modified Tegner scale^a^	8.0 (2.0)	8.0 (2.0)	.789	-.04
Participating level, n (%)			.294	.25
Recreation	4 (25)	2 (7)		
Competition	9 (56)	20 (71)		
Elite	3 (19)	6 (22)		

BMI, body mass index; BTB, bone-patellar tendon-bone; NRTS, no-return to sports; PoSAP, postoperative subjective athletic performance; ST, semitendinosus; YRTS, yes-return to sports.

a Median (interquartile range), ∗*P* < .05.

The knee function and landing impact data are shown in Table 2. The NRTS group had a lower LSI of knee extensor strength at 180°/s (*P* = .032, *d* = .18) and pVGRF magnitude (*P* = .019, *d* = .77) than the YRTS group. The results of Fisher's exact test (Table 3) showed that patients in the NRTS group (50%) had a higher rate of pVGRF asymmetry than patients in the YRTS group (18%) (*P* = .040, *V* = .34).

**Table 2:** Group differences in knee function and the parameters of ground reaction force during single-leg jump landing.

	Variables	NRTS (n = 16)	YRTS (n = 28)	*P* value	Effect size
Knee ROM	Extension ROM deficit, deg^a^	.0 (1.0)	0.0 (1.8)	.747	-.05
	Flexion ROM deficit, deg^a^	0.0 (5.0)	0.0 (0.0)	.136	-.23
Knee joint effusion	Stroke test grading Zero, n	13	25	.582	.21
	Trace, n	2	3		
	1+, n	1	0		
Knee laxity	Difference of maximum pull, mm, mean ± SD	1.0 ± 1.4	1.0 ± 1.3	.934	.03
Knee strength	Extension LSI, 60°/s, %, mean ± SD	87.1 ± 10.9	91.6 ± 11.7	.215	.39
	Flexion LSI, 60°/s %, mean ± SD	92.6 ± 11.7	92.7 ± 11.0	.990	.01
	Extension LSI, 180°/s %, mean ± SD	86.8 ± 7.4	92.1 ± 7.9	.032∗	.18
	Flexion LSI, 180°/s %, mean ± SD	91.5 ± 11.1	93.9 ± 9.8	.461	.23
Single-leg hop for distance	Hop distance LSI, %^a^	97.2 (7.9)	99.6 (6.8)	.532	-.09
Landing impact	pVGRF magnitude on operated side, %BW, mean ± SD	330.0 ± 39.2	365.6 ± 49.9	.019∗	.77

BW, body weight; LSI, limb symmetry index; NRTS, no-return to sports; pVGRF, peak vertical ground reaction force; ROM, range of motion; SD, standard deviation; YRTS, yes-return to sports.

a Median (interquartile range), ∗*P* < .05.

**Table 3:** Comparison of RTS status and pVGRF asymmetry during single-leg jump landing.

		NRTS	YRTS	Total
pVGRF asymmetry	No	8	23	31
	Yes	8	5	13
	Total	16	28	44

VGRF asymmetry was defined as ASI >10%. ASI, absolute symmetry index; NRTS, no-return to sports; YRTS, yes-return to sports; pVGRF, peak vertical ground reaction force.

The results of the logistic regression analysis to determine the association between landing impact and the RTS status are shown in Table 4. In model 1, both pVGRF magnitude and asymmetry were significantly associated with the RTS status. In model 2 (adjusted for covariates), pVGRF magnitude was the only factor significantly associated with the RTS status. To account for multicollinearity and the correct classification rate of regression analysis, BMI was used as a continuous variable.

**Table 4:** Logistic regression analysis to determine the association between landing impact and RTS status.

Model 1				
Independent variable	*P* value	Odds ratio	95% confidence interval	
			Lower	Upper
pVGRF magnitude on operated side	.022	0.980	0.964	0.997
pVGRF asymmetry	.024	6.036	1.271	28.656
χ2 test, *P* < .001; Hosmer-Lemeshow test, *P* = .099; percentage of correct classifications, 72.7%.				

Model 2				
Independent variable	*P* value	Odds ratio	95% confidence interval	
			Lower	Upper
pVGRF magnitude on operated side	.015	0.974	0.953	0.995
pVGRF asymmetry	.203	3.186	0.534	19.007
Extension LSI, 180°/s	.154	0.909	0.797	1.036
BMI	.298	1.174	0.868	1.588

χ2 test, *P* = .002; Hosmer-Lemeshow test, *P* = .681; percentage of correct classifications, 72.7%.

Model 2: Model 1 + covariance (extension LSI (180°/s), BMI).

pVGRF asymmetry was defined as ASI >10%.

ASI, absolute symmetry index; BMI, body mass index; LSI, limb symmetry index; pVGRF, peak vertical ground reaction force.

## Discussion

The present study investigated the association between the RTS status and VGRF parameters during single-leg jump landing in post-ACLR patients. Our analysis showed that the pVGRF magnitude on operated side during single-leg jump landing was associated with the RTS status, even after adjusting for the LSI of knee extensor strength and BMI. These results partially support our hypothesis and provide new data showing that pVGRF magnitude during single-leg jump landing is associated with the RTS status.

Logistic regression analysis showed that the pVGRF magnitude was associated with the RTS status (Table 4, model 2). The VGRF of the eccentric deceleration phase during single-leg vertical jumping is smaller in post-ACLR patients with lower International Knee Documentation Committee (IKDC) scores than in patients with higher IKDC scores.^30^ In post-ACLR patients, IKDC is a representative subjective outcome of knee symptoms during sporting activities.^31^ Although the task and outcome in the present study were different, this supports the results of previous studies that the pVGRF of individuals with low scores on subjective outcomes in sporting activities, including knee function, is smaller than that of individuals with high scores.

Since we did not include healthy athletes, it is difficult to interpret the magnitude of the relationship of pVGRF in the YRTS and NRTS groups solely based on the results. A previous study of healthy athletes measured VGRF during single-leg jump landing with the same equipment and method, and the median (interquartile range) pVGRF was 371.7 (83.1) %body weight.^27^ Although the results of this study cannot be statistically compared with those of previous studies, the results suggest that the pVGRF of the YRTS group is not larger than that of healthy athletes, but that the pVGRF of the NRTS group is smaller.

In the single-leg jump landing task, post-ACLR patients showed a different landing strategy than healthy athletes. Pozzi et al.^32^ showed that in patients after ACLR, the moment required to support the centre of gravity during a single-leg drop landing is relatively greater in the hip than in the knee, compared to healthy participants. Vairo et al.^33^ showed that post-ACLR patients had a smaller pVGRF and greater hip flexion angle during single-leg jump landing than healthy athletes. Hip flexion significantly contributes to landing force absorption^34^ and consciously flexed lower extremity joints while landing reduced the pVGRF.^35^ Excessive pVGRF increases ACL load, indicating that post-ACLR patients may avoid loading their knee during landing.^10^^,^^11^ Our results suggest that such compensatory movements may have occurred, particularly among the NRTS group's patients whose performance was inadequate.

We observed that a higher proportion of patients in the NRTS group showed asymmetrical pVGRF than those in the YRTS group (Table 4, model 1). Post-ACLR patients with lower IKDC scores had a greater pVGRF asymmetry during single-leg landing than patients with higher IKDC scores.^13^ Post-ACLR patients with decreased knee extensor strength on the operated side had smaller pVGRF LSIs during landing than those who did not have decreased knee extensor strength.^36^ Landing impact asymmetry is related to poor subjective and objective knee function related to RTS in post-ACLR patients; therefore, it is important to correct it.

The NRTS group had a higher BMI and proportion of overweight individuals than the YRTS group. A systematic review examining BMI in post-ACLR patients showed that overweight individuals had an increased risk of secondary problems such as arthritis and osteoarthritis than those who were not overweight.^37^ The results of this study provide new data on the relationship between BMI and the RTS status. However, patients in some sports or weight categories need to maintain a high weight. Therefore, careful interpretation of BMI is necessary.

Our study showed that the LSI of knee extensor strength in the NRTS group was smaller than that of the YRTS group. Our results are consistent with a previous study^6^ that showed a lower LSI of knee extensor strength in the NRTS group than in the YRTS group. Schmitt^36^ showed that post-ACLR patients with a low extensor strength LSI had smaller pVGRF and pVGRF LSI on the affected side than those with a high extensor strength LSI. Thus, postoperative knee extensor strength is an important functional variable related to RTS and landing impact, and its improvement is vital during rehabilitation.

The present study did not find associations between the RTS status and functional variables, such as ROM, laxity, effusion, flexor strength, and single-leg hop for distance, because the proportion of functional impairment on the non-operated side was low. These functional variables become worse after an ACL injury or ACLR and are the criteria for sports participation.^38^ Associations between these variables and the RTS status have previously been demonstrated, and they remain important knee function variables. Our results provide evidence that even when these knee function variables improve, the landing impact during single-leg jump landing is associated with the RTS status.

### Clinical implications

Post-ACLR patients not only have a lower pVGRF during single-leg landings compared with both their non-operated side and healthy athletes, but also their kinetic pattern includes a lower knee internal extension moment and a comparatively greater moment of the hip joint than of the knee.^32^ Post-ACLR patients also show specific kinematic patterns, such as greater trunk and hip flexion angles during single-leg landing compared with both their non-operated side and healthy athletes.^33^^,^^39^ These patterns are regarded as compensatory movements to avoid loading the knee, and it has been suggested that the pVGRF in the NRTS group may be related to these kinetic and kinematic patterns. In particular, these patterns are more likely to be found in those with weak extensor muscle strength,^39^ and the results were similar in the present study. Based on these findings, patients in the NRTS group may require the following instructions to RTS at their pre-injury competition level: 1) correct knee extensor strength asymmetry and 2) check and correct kinetic and kinematic patterns to enable them to tolerate a landing impact on the operated side that is both within the normal range and close to the impact on the non-operated side to correct its asymmetry. However, excessive pVGRF is a risk factor for ACL injury^8^ and patients must be taught to land while keeping it within the reference values determined in healthy adults.

### Study limitations

We could not evaluate causal associations because of the cross-sectional design. Future cohort studies are needed to determine the impact of VGRF characteristics during landing on future RTS. The pVGRF is a representative variable for assessing the landing impact; however, landing impact changes with the foot, knee, hip, and trunk movements.^34^ Future analysis of the kinetics/kinematics of these joints, in addition to pVGRF, may elucidate landing strategies associated with the RTS status, enabling the provision of appropriate landing instructions. The pVGRF may differ between dominant and non-dominant limb, but these effects were not taken into account in this study. The impact characteristics differ per the type of task. In the single-leg jump landing task, we measured the impact when the participants landed in whatever way they prefer. However, tasks such as jumping again after landing, which assesses the energy production capacity, and soft landing, which assesses absorption capacity,^35^ were not evaluated. Further studies comparing multiple landing tasks are required to ascertain more specific functions of athletic performance. Participants in this study had a wide range of post-operative follow-up (24.8 ± 18.4 months). We cannot discount the possibility that functional impairment, including impairment on the nonoperative side, may occur in the elapsed years.^40^ Finally, postoperative psychology such as kinesiophobia is associated with RTS status^5^ and landing biomechanics,^41^ but these effects are unknown in this study.

## Conclusion

We examined the associations between the RTS status and the magnitude and asymmetry of landing impact during single-leg jump landing in post-ACLR patients. The pVGRF of post-ACLR patients who were unable to RTS at their pre-injury competition level was lower and more asymmetrical than patients who had returned to their pre-injury competition level. The magnitude of pVGRF was associated with the RTS status, even after adjusting for other knee function variables. Landing practice to enable the patients to tolerate the landing impact and instructions regarding asymmetry correction may be important for supporting post-ACLR patients in their RTS.

## Funding

None declared.

## Declaration of competing interest

None declared.

## References

[1] Feucht M.J., Cotic M., Saier T. (2016). Patient expectations of primary and revision anterior cruciate ligament reconstruction. Knee Surg Sports Traumatol Arthrosc.

[2] Ardern C.L., Webster K.E., Taylor N.F., Feller J.A. (2011). Return to sport following anterior cruciate ligament reconstruction surgery: a systematic review and meta-analysis of the state of play. Br J Sports Med.

[3] Czuppon S., Racette B.A., Klein S.E., Harris-Hayes M. (2014). Variables associated with return to sport following anterior cruciate ligament reconstruction: a systematic review. Br J Sports Med.

[4] Webster K.E., Feller J.A. (2018). Return to level I sports after anterior cruciate ligament reconstruction: evaluation of age, sex, and readiness to return criteria. Orthop J Sports Med..

[5] Lentz T.A., Zeppieri G., Tillman S.M. (2012). Return to preinjury sports participation following anterior cruciate ligament reconstruction: contributions of demographic, knee impairment, and self-report measures. J Orthop Sports Phys Ther.

[6] Lentz T.A., Zeppieri G., George S.Z. (2015). Comparison of physical impairment, functional, and psychosocial measures based on fear of reinjury/lack of confidence and return-to-sport status after ACL reconstruction. Am J Sports Med.

[7] Webster K.E., McPherson A.L., Hewett T.E., Feller J.A. (2019). Factors associated with a return to preinjury level of sport performance after anterior cruciate ligament reconstruction surgery. Am J Sports Med.

[8] Hewett T.E., Myer G.D., Ford K.R. (2005). Biomechanical measures of neuromuscular control and valgus loading of the knee predict anterior cruciate ligament injury risk in female athletes: a prospective study. Am J Sports Med.

[9] Dai B., Butler R.J., Garrett W.E., Queen R.M. (2014). Using ground reaction force to predict knee kinetic asymmetry following anterior cruciate ligament reconstruction. Scand J Med Sci Sports.

[10] Lepley A.S., Kuenze C.M. (2018). Hip and knee kinematics and kinetics during landing tasks after anterior cruciate ligament reconstruction: a systematic review and meta-analysis. J Athl Train.

[11] Nyland J., Klein S., Caborn D.N. (2010). Lower extremity compensatory neuromuscular and biomechanical adaptations 2 to 11 years after anterior cruciate ligament reconstruction. Arthroscopy.

[12] Renner K.E., Franck C.T., Miller T.K., Queen R.M. (2018). Limb asymmetry during recovery from anterior cruciate ligament reconstruction. J Orthop Res.

[13] Baumgart C., Schubert M., Hoppe M.W., Gokeler A., Freiwald J. (2017). Do ground reaction forces during unilateral and bilateral movements exhibit compensation strategies following ACL reconstruction?. Knee Surg Sports Traumatol Arthrosc.

[14] Heebner N.R., Rafferty D.M., Wohleber M.F. (2017). Landing kinematics and kinetics at the knee during different landing tasks. J Athl Train.

[15] Koga H., Nakamae A., Shima Y. (2010). Mechanisms for noncontact anterior cruciate ligament injuries: knee joint kinematics in 10 injury situations from female team handball and basketball. Am J Sports Med.

[16] Johnston P.T., McClelland J.A., Webster K.E. (2018). Lower limb biomechanics during single-leg landings following anterior cruciate ligament reconstruction: a systematic review and meta-analysis. Sports Med.

[17] Fältström A., Hägglund M., Kvist J. (2013). Patient-reported knee function, quality of life, and activity level after bilateral anterior cruciate ligament injuries. Am J Sports Med.

[18] Ohji S., Aizawa J., Hirohata K. (2020). The gap between subjective return to sports and subjective athletic performance intensity after anterior cruciate ligament reconstruction. Orthopaedic Journal of Sports Medicine.

[19] Brosseau L., Balmer S., Tousignant M. (2001). Intra- and intertester reliability and criterion validity of the parallelogram and universal goniometers for measuring maximum active knee flexion and extension of patients with knee restrictions. Arch Phys Med Rehabil.

[20] Sturgill L.P., Snyder-Mackler L., Manal T.J., Axe M.J. (2009). Interrater reliability of a clinical scale to assess knee joint effusion. J Orthop Sports Phys Ther.

[21] Brosky J.A., Nitz A.J., Malone T.R., Caborn D.N., Rayens M.K. (1999). Intrarater reliability of selected clinical outcome measures following anterior cruciate ligament reconstruction. J Orthop Sports Phys Ther.

[22] Robnett N.J., Riddle D.L., Kues J.M. (1995). Intertester reliability of measurements obtained with the KT-1000 on patients with reconstructed anterior cruciate ligaments. J Orthop Sports Phys Ther.

[23] Muneta T., Koga H., Ju Y.J., Yagishita K., Sekiya I. (2011). Effects of different initial bundle tensioning strategies on the outcome of double-bundle ACL reconstruction: a cohort study. Sports Med Arthrosc Rehabil Ther Technol.

[24] Aizawa J., Ohji S., Hirohata K., Ohmi T., Koga H., Yagishita K. (2019). Relationship between asymmetrical jump-landing impact and quadriceps strength after unilateral anterior cruciate ligament reconstruction. Phys Med.

[25] Fitzgerald G.K., Lephart S.M., Hwang J.H., Wainner R.S. (2001). Hop tests as predictors of dynamic knee stability. J Orthop Sports Phys Ther.

[26] Ohji S., Aizawa J., Hirohata K. (2021). Single-leg hop can result in higher limb symmetry index than isokinetic strength and single-leg vertical jump following anterior cruciate ligament reconstruction. Knee.

[27] Ohji S., Aizawa J., Hirohata K., Ohmi T., Yagishita K. (2019). Correlations between vertical ground reaction force, sagittal joint angles, and the muscle Co-contraction index during single-leg jump-landing. Asian J Sports Med.

[28] Aizawa J., Ohji S., Koga H., Masuda T., Yagishita K. (2016). Correlations between sagittal plane kinematics and landing impact force during single-leg lateral jump-landings. J Phys Ther Sci.

[29] Wang J., Fu W. (2019). Asymmetry between the dominant and non-dominant legs in the lower limb biomechanics during single-leg landings in females. Adv Mech Eng.

[30] Baumgart C., Hoppe M.W., Freiwald J. (2017). Phase-specific ground reaction force analyses of bilateral and unilateral jumps in patients with ACL reconstruction. Orthop J Sports Med..

[31] Shelbourne K.D., Barnes A.F., Gray T. (2012). Correlation of a single assessment numeric evaluation (SANE) rating with modified Cincinnati knee rating system and IKDC subjective total scores for patients after ACL reconstruction or knee arthroscopy. Am J Sports Med.

[32] Pozzi F., Di Stasi S., Zeni J.A., Barrios J.A. (2017). Single-limb drop landing biomechanics in active individuals with and without a history of anterior cruciate ligament reconstruction: a total support analysis. Clin Biomech.

[33] Vairo G.L., Myers J.B., Sell T.C., Fu F.H., Harner C.D., Lephart S.M. (2008). Neuromuscular and biomechanical landing performance subsequent to ipsilateral semitendinosus and gracilis autograft anterior cruciate ligament reconstruction. Knee Surg Sports Traumatol Arthrosc.

[34] Devita P., Skelly W.A. (1992). Effect of landing stiffness on joint kinetics and energetics in the lower extremity. Med Sci Sports Exerc.

[35] Blackburn J.T., Padua D.A. (2009). Sagittal-plane trunk position, landing forces, and quadriceps electromyographic activity. J Athl Train.

[36] Schmitt L.C., Paterno M.V., Ford K.R., Myer G.D., Hewett T.E. (2015). Strength asymmetry and landing mechanics at return to sport after anterior cruciate ligament reconstruction. Med Sci Sports Exerc.

[37] DiSilvestro K.J., Jauregui J.J., Glazier E. (2019). Outcomes of anterior cruciate ligament reconstruction in obese and overweight patients: a systematic review. Clin J Sport Med.

[38] Bousquet B.A., O'Brien L., Singleton S., Beggs M. (2018). POST-OPERATIVE criterion based rehabilitation OF ACL repairs: a clinical commentary. Int J Sports Phys Ther..

[39] Ithurburn M.P., Paterno M.V., Ford K.R., Hewett T.E., Schmitt L.C. (2015). Young athletes with quadriceps femoris strength asymmetry at return to sport after anterior cruciate ligament reconstruction demonstrate asymmetric single-leg drop-landing mechanics. Am J Sports Med.

[40] Patterson B.E., Crossley K.M., Perraton L.G. (2020). Limb symmetry index on a functional test battery improves between one and five years after anterior cruciate ligament reconstruction, primarily due to worsening contralateral limb function. Phys Ther Sport.

[41] Trigsted S.M., Cook D.B., Pickett K.A., Cadmus-Bertram L., Dunn W.R., Bell D.R. (2018). Greater fear of reinjury is related to stiffened jump-landing biomechanics and muscle activation in women after ACL reconstruction. Knee Surg Sports Traumatol Arthrosc.

